# The germline variants in DNA repair genes in pediatric *medulloblastoma*: a challenge for current therapeutic strategies

**DOI:** 10.1186/s12885-017-3211-y

**Published:** 2017-04-04

**Authors:** Joanna Trubicka, Tomasz Żemojtel, Jochen Hecht, Katarzyna Falana, Dorota Piekutowska- Abramczuk, Rafał Płoski, Marta Perek-Polnik, Monika Drogosiewicz, Wiesława Grajkowska, Elżbieta Ciara, Elżbieta Moszczyńska, Bożenna Dembowska-Bagińska, Danuta Perek, Krystyna H. Chrzanowska, Małgorzata Krajewska-Walasek, Maria Łastowska

**Affiliations:** 1 Department of Medical Genetics, The Children’s Memorial Health Institute, Al. Dzieci Polskich 20, 04-730 Warsaw, Poland; 2 Department of Pathology, The Children’s Memorial Health Institute, Al. Dzieci Polskich 20, 04-730 Warsaw, Poland; 3 Institute for Medical Genetics and Human Genetics, Charité Universitätsmedizin Berlin, Augustenburger Platz 1, 13353 Berlin, Germany; 4 Institute of Bioorganic Chemistry, Polish Academy of Sciences, 60-569 Poznan, Poland; 5 Max Planck Institute for Molecular Genetics, Ihnestr. 63-73, 14195 Berlin, Germany; 6 Berlin-Brandenburg Center for Regenerative Therapies, Charité Universitätsmedizin Berlin, 13353 Berlin, Germany; 7 Department of Medical Genetics, Warsaw Medical University, Warsaw, Poland; 8 Department of Oncology, The Children’s Memorial Health Institute, Al. Dzieci Polskich 20, 04-730 Warsaw, Poland; 9 Department of Experimental and Clinical Pathology, Mossakowski Medical Research Centre Polish Academy of Sciences, A. Pawińskiego 5, 02-106 Warsaw, Poland; 10 Department of Endocrinology and Diabetology, The Children’s Memorial Health Institute, Al. Dzieci Polskich 20, 04-730 Warsaw, Poland

**Keywords:** Medulloblastoma, DNA repair genes, Toxicity

## Abstract

**Background:**

The defects in DNA repair genes are potentially linked to development and response to therapy in *medulloblastoma*. Therefore the purpose of this study was to establish the spectrum and frequency of germline variants in selected DNA repair genes and their impact on response to chemotherapy in *medulloblastoma* patients.

**Methods:**

The following genes were investigated in 102 paediatric patients: *MSH2* and *RAD50* using targeted gene panel sequencing and *NBN* variants (p.I171V and p.K219fs*19) by Sanger sequencing. In three patients with presence of rare life-threatening adverse events (AE) and no detected variants in the analyzed genes, whole exome sequencing was performed. Based on combination of molecular and immunohistochemical evaluations tumors were divided into molecular subgroups. Presence of variants was tested for potential association with the occurrence of rare life-threatening AE and other clinical features.

**Results:**

We have identified altogether six new potentially pathogenic variants in *MSH2* (p.A733T and p.V606I), *RAD50* (p.R1093*), *FANCM* (p.L694*), *ERCC2* (p.R695C) and *EXO1* (p.V738L), in addition to two known *NBN* variants. Five out of twelve patients with defects in either of *MSH2*, *RAD50* and *NBN* genes suffered from rare life-threatening AE, more frequently than in control group (*p* = 0.0005). When all detected variants were taken into account, the majority of patients (8 out of 15) suffered from life-threatening toxicity during chemotherapy.

**Conclusion:**

Our results, based on the largest systematic study performed in a clinical setting, provide preliminary evidence for a link between defects in DNA repair genes and treatment related toxicity in children with *medulloblastoma*. The data suggest that patients with DNA repair gene variants could need special vigilance during and after courses of chemotherapy.

**Electronic supplementary material:**

The online version of this article (doi:10.1186/s12885-017-3211-y) contains supplementary material, which is available to authorized users.

## Background

Brain tumors represent the leading cause of childhood cancer mortality. The most common malignant brain tumor among them is *medulloblastoma* [1]. Although multimodality treatment regimens have substantially improved survival in this disease, up to 30-40% of patients with *medulloblastoma* still die of the disease. Detrimental effect of current treatment on long-term survivors is also observed [2]. Our understanding of the molecular background of pediatric brain tumors has expanded significantly over the past few years. The vast amount of genomic and molecular data generated recently has proved that *medulloblastoma* is not a single entity but is composed of at least four subtypes: Wingless (WNT), Sonic Hedgehog (SHH), Group 3 and Group 4 (non-WNT/SHH types), with distinct genetic and biological profiles as well as different course of disease requiring adequate therapeutic approaches [2–6]. Despite of improved understanding of the molecular basis of *medulloblastoma*, many cases still lack an obvious genetic driver [4, 7, 8]. The further research focused on additional potential mechanisms responsible for the development of this tumor may lead to identification of new susceptibility factors as well as new markers that predict response to therapeutic agents and provide prognostic information. So far, majority of driver mutations detected in *medulloblastoma* are of somatic character. Impact of germline genetic variability that may affect clinicopathologic presentation of this tumor have not been in-depth investigated yet [5, 7, 9–12].

In our study we focused on evaluation of germline defects in genes that play a role in DNA repair pathway because of the following reasons. Firstly, DNA-repair deficiency is associated with cancer development and the key role of germline alterations in promoting tumorigenesis is highlighted by several cancer predisposition syndromes e.g. Li-Fraumeni, Fanconi anemia or Turcot syndrome, where occurrence of *medulloblastoma* has been recorded. Secondly, it is well known that germline defects may modulate the response to treatment since DNA-repair mechanisms make cells prone to the effects of DNA-damaging chemotherapy [13–15]. It is important to notice that majority of evidence about the impact of DNA-repair genes defects on toxicity in *medulloblastoma* comes from either description of single cases [16, 17] or from mouse models and cell lines experiments [13, 18] but not from systemic clinical based investigation. Therefore, all these data indicate that DNA repair genes are a promising targets possibly linked both to development of tumor and response to therapy in *medulloblastoma*.

Within essential components of DNA repair signaling cascade the *NBN* gene particularly draws attention as potentially susceptibility marker for *medulloblastoma* [19, 20]. Germline defects in *medulloblastoma* patients were observed also in other genes cooperated with *NBN* in BRCA1-associated genome surveillance complex (BASC), including *MSH6*, *PMS2* and *MLH1* [21–24].

Biallelic defects in *NBN* gene result in Nijmegen Breakage Syndrome (NBS; OMIM:251,260), while homozygous defects in *MSH6*, *PMS2* or *MLH1* genes are molecular cause of Constitutional Mismatch Repair Deficiency Syndrome (CMRDS; OMIM:276,300), hereditary disorder associated with increased risk of cancers including *medulloblastoma* [25]. Among other genes responsible for CMRDS is also *MSH2* (ID:4436, MIM:609,309), one of the key factor of DNA mismatch repair system which recognizes and repairs mispaired or unpaired nucleotides resulted from DNA replication errors [25]. There is an evidence that germline *MSH2* defects may predispose to primary early-onset CNS tumors, especially *glioblastoma* [26]. In addition, De Rosa et al. suggest that in some families with Turcot syndrome the coexistence of colorectal and childhood brain tumors may result from a complete MMR deficiency [27]. However, association between *MSH2* defects and *medulloblastoma* was not evaluated yet.

A very similar phenotype to NBS was seen in patients with Nijmegen Breakage Syndrome-like Disorder (NBSLD – OMIM:613,078) caused by defects in the *RAD50* gene (ID:10,111, MIM:604,040). This gene encodes the protein involved in DNA double-strand break repair, cell cycle checkpoint activation, telomere maintenance and meiotic recombination suggesting that molecular variants disrupting its function may lead to genome instability and carcinogenesis [28]. Furthermore, inactivation of proteins like RAD50 required for the homologous recombination machinery leads to defects in the nervous system development indicating that components of this system can play crucial role in development and progression of various neuro-oncological diseases [29]. The frequency of the molecular variants in *RAD50* gene was, similarly to *MSH2*, not determined in *medulloblastoma* patients up to now.

Therefore the first purpose of this study was to establish the spectrum of germline defects in *MSH2* and *RAD50* genes, as well as frequency of two known *NBN* variants in 102 patients with *medulloblastoma*. In the next step we have evaluated the hypothesis that DNA repair genes may affect a response to therapy in *medulloblastoma* patients. We have found that alterations in a range of DNA repair genes are associated with occurrence of rare severe adverse effects during chemotherapy in patients.

## Methods

### Patients and controls

A set of 102 *medulloblastoma* patients treated between 2004 and 2014 in the Neurosurgery and Oncology Departments of the Children’s Memorial Health Institute (CMHI) in Warsaw, Poland were investigated in this study. Based on a combination of genomic and immunohistochemical (IHC) analyses, patients were divided into molecular subgroups (see methods). Presence of metastases at diagnosis was classified according to Chang et al. [30]. The clinical characteristics of the study cohort is outlined in Table 1.

**Table 1:** The characteristics of 102 patients with *medulloblastoma*

*Medulloblastoma* patients		Number of patients
Age at diagnosis (years)	≤ 3	14
	3-13	88
Gender	Male	66
	Female	36
Histologic type	Classic	69
	LCA	16
	D/N	6
	MBEN	6
	MBL	5
Metastasis	NO	70
	YES	31
	NA	1
Molecular tumor subtype	WNT	11
	SHH	9
	Non-WNT/SHH	25
	Group 3	7
	Group 4	24
	NA	26
Treatment protocol	HR	60
	SR	29
	<3 yrs	13
	TOTAL	102

Abbreviations: *LCA* large cell/anaplastic, *D/N* desmoplastic/nodular, *MBEN* with extensive nodularity, *MBL medulloblastoma*, subtype not known, *NA* not available, *PPNG* Polish Pediatric Neurooncology Group, *HR -PPNG* High Risk protocol, *SR* -*PPNG* Standard Risk protocol, <3 yrs. – PPNG protocol for children younger than 3 years old

To estimate the population frequency of detected *MSH2* and *RAD50* variants (independently of the data deposited in the public databases) the population-specific control group was assembled. DNA samples from 300 healthy donors with negative cancer family history and sex matched to the patients’ groups were collected. To exclude potential bias between adult and childhood population the control group consisted of donors age matched to the study group.

### Methods

A total DNA was extracted from peripheral blood and tumors samples by use of the automatic magnetic bead-based (MagnaPure, Roche) and phenol/chloroform methods, respectively [31].

To evaluate the sequence of *MSH2* and *RAD50* genes in 102 patients with *medulloblastoma* targeted gene panel sequencing was used. The *NBN* c.511A>G and c.657_661del5 variant status was determined upon previously described conditions [19]. In three patients with severe adverse events after the chemotherapy but with no variants detected in *MSH2*, *RAD50,* and *NBN* genes whole exome sequencing (WES) was carried out to explore possible defects in other DNA repair genes.

#### Targeted gene panel sequencing

For generation of the targeted amplicon libraries the Ion AmpliSeq™ Custom 3G-Panelv2 (275 bp; Life Technologies Corporation; Carlsbad, CA, USA) consisting of 82 primer pairs to target all exons of the *MSH2* and *RAD50* genes (RefSeq:NM_000251.2 and RefSeq: NM_005732.3, respectively) was used. Polymerase chain reaction (PCR) was performed according to the manufacturer’s recommendations with the Ion AmpliSeq™ Library Kit 2.0. Amplicon size distribution and library concentration was determined using Agilent DNA 1000 Kit (Agilent Technologies; Inc., Waldbronn, Germany). The final concentration of the sample pool was measured by Qubit dsDNA BR Assay Kit (Life Technologies Corporation; Carlsbad, CA, USA). Emulsion PCR and sequencing was performed on an Ion PGM Sequencer (Life Technologies Corporation; Carlsbad, CA, USA) using 318 Chips and the Ion PGM 200 Sequencing Kit according to the manufacturer’s instructions. The sequence reads were mapped to the haploid human reference genome (hg19) with Novoalign (Novocraft Technologies). SNVs and short insertions and deletions (indels) were called using GATK version 2.8. [32] Variant annotation was performed with Jannovar [33].

#### Whole exome sequencing

WES Library preparation was performed using Nextera Rapid Capture Exome kit (Illumina). The samples were run on ¼ of lane each on HiSeq 1500 using 2 × 75 bp paired-end reads. After initial processing by the CASAVA, the generated reads were aligned to the hg19 reference genome with Burrows-Wheeler Alignment Tool and further processed by Genome Analysis Toolkit [32]. Base quality score recalibration, indel realignment, duplicate removal and the SNP/INDEL calling were done as described [34]. The detected variants were annotated using Annovar5 [35].

#### Selection and validation of candidate variants

A way of prioritizing variants was based on three main filtration steps, as follows:

1. variants with the global minor allele frequency (MAF) ≥ 0.01 (either in the 1000 Genomes Project, ExAC Databases or in a matched control group) were filtered out;
2. nonsynonymous SNPs, coding sequence insertion/deletions (indels), and canonical splice-site variants were selected;
3. all variants occurring within DNA repair genes detected by WES were prioritized;
4. variants annotated as deleterious by three and more prediction algorithms (PolyPhen-2, SIFT, Mutation Taster and FATHMM), nonsense mutations and deletions that introduce premature stop codons were classified as likely pathogenic and selected into further consideration. Functional consequences of splice variants were predicted by Human Splicing Finder, Splice Site Finder-like and MaxEntScan.

For all selected variants the amino acid position in functional domains and posttranslational modifications were verified using NCBI Protein [36] and Alamut-2.4-6 Software (Interactive Biosoftware; Rouen, France). Their contribution in carcinogenesis was verified in the Catalogue of Somatic Mutations (COSMIC database) [37], Human Gene Mutation Database (HGMD) [38], ClinVar database and Online Mendelian Inheritance in Man (OMIM). Correlation with the clinical features and the course of disease was assessed for each candidate variant.

#### Variant validation

Next generation sequencing results were validated using Sanger sequencing. Specific primers for PCR reactions are available upon request. PCR products were sequenced in 3130 Genetic Analyzer and evaluated with Sequencing Analysis Software v.5.4 (Applied Biosystems/Life Technologies; Foster City, CA). The frequency of validated variants were evaluated in the matched control group in the same conditions. Additionally, prioritized variants in *MSH2* and *RAD50* were cross-checked with the parents’ sequence data to identify inherited versus de novo changes. In four out of five carriers of *MSH2*, *RAD50*, *FANCM* or *EXO1* gene variants tumour tissues were available and the presence of identified changes (p.V606I, p.R1093*, p.L694* and p.V738L) were verified.

#### Determination of molecular subgroups in medulloblastoma patients

Tumors included in the study were divided into the following molecular groups:

1. WNT Group defined by the presence of at least two features as recommended by the International *Medulloblastoma* Working Group [2]: *CTNNB1* pathogenic variant, immunohistochemical positive nuclear reaction against β-catenin (DB #610154, 1:800) and the presence of chromosome 6 monosomy. The screening test for *CTNNB1* variant analysis and chromosome 6 monosomy were performed according to methods described previously [39].
2. SHH Group defined by the presence of immunohistochemical positive reaction with anti-GAB1 (Abcam #ab27439 and/or #59362, 1:100) and anti-YAP1 (Santa Cruz #sc-101,199, 1:50) antibodies [3].
3. Non-WNT/SHH type (Group 3 or Group 4) included the remaining tumors, tested negative for the above features.
4. For further discrimination of non-WNT/SHH tumours (in order to identify Group 3 or Group 4) in patients with detected DNA repair genes variants we applied NanoString's nCounter System analysis (NanoString Technologies, Seattle, USA). For identification of clusters a series of 48 *medulloblastoma* tumours were analized. Overall 30 non-WNT/SHH tumours from this study were evaluated. Total RNA was extracted from frozen or FFPE tumours using RNeasy kits (Qiagen). RNA integrity was assessed using an Agilent 2100 bioanalyzer. For four Groups assignment NanoString CodeSet of 22 genes has been applied as described by Northcott et al. [40] Hybridization to the probes was performed in NanoString Technologies, Seattle, USA. Data were normalized and samples were clustered using nSolver 2.5 software.

#### Treatment complications assessment

Treatment related complications data were retrospectively re-analyzed and assessed according to The Common Toxicity Criteria (CTC), version 4.0. [41] For comparative analyses only rare life-threatening adverse events of grade 4 (grade 5 was absent in our cohort) have been taken into account since they are the most challenging complications in clinical practice. The significance of assessed adverse events frequency and other clinical features in groups of patients with presence vs. absence of DNA repair genes candidate variants were calculated using the Fisher Exact test.

## Results

### *MSH2* and *RAD50* analysis

The analysis of *MSH2* and *RAD50* coding sequences in 102 *medulloblastoma* patients revealed 53 germline variants in total. Most of them (48/53; 90.6%) were single nucleotide variants (SNV), while remaining (5/53; 9.4%) were small indels (Additional file 1: Table S1). The number of identified variants ranged from 2 to 16, an average of 7 variants per sample. After the filtration steps (three heterozygous candidate variants including c.1816G>A (p.V606I) and c.2197G>A (p.A733T) in *MSH2*, as well as c.3277C>T (p.R1093*) in *RAD50* were selected. All of them were uncommon (1/102; 0.98% for each variant) in the patients group. None of them was observed in 1000 Genomes Project Database [42] as well as in the matched control group of 300 samples. Allele frequency of p.A733T *MSH2* and p.R1093* *RAD50* variants in ExAC Database were 0.000005 and 0.000001, respectively. The *MSH2* p.V606I variant in ExAC Database was not reported so far (Table 2).

**Table 2:** Germline candidate variants detected in the current study in DNA repair genes in *medulloblastoma* patients

Variant	Gene	Effect on gene function	Wild type nucleotide	Altered nucleotide	Frequency in matched control group	Frequency in 1000 Genomes Database	Frequency in ExAC Database	“In silico” analysis of pathogenicity^a^				High conserved nucleotide	Protein domain	ClinVar
								PolyPhen-2	SIFT	FATHMM	Mutation Taster			
Missense, nonsense mutation and deletion														
p.V606I	*MSH2*	missense	G	A	0	0	0	P score 0.868	T score 0.09	D score − 2.61	D score 0.9999	yes phyloP: 0.91	1.DNA mismatch repair protein MutS, 2.DNA mismatch repair protein MSH2	no data
p.A733T	*MSH2*	missense	G	A	0	0	0.000005	D score 0.985	D score 0	D score − 2.3	D score 0.9999	yes phyloP: 0.91	1.DNA mismatch repair protein MutS 2.P-loop containing nucleotide triphosphate hydrolase 3.DNA mismatch repair protein MSH2	no data
p.I171V	*NBN*	missense	A	G	0	0.001	0.00013	D score 1.000	D score 0	T score 1	D score 0.9999	yes phyloP: 0.96	1.BRCT domain 2.nibrin	RCV000007360.2 RCV000007361.2 RCV000115797.5
p.L219Afs	*NBN*	deletion	ACAAA	0	0	0	0.000019	-	-	-	D score 0.9999	-	1.nibrin	RCV000133576.3 RCV000007353.2
p.R695C	*ERCC2*	missense	G	A	0	0	0.000000012	D score 0.995	D score 0	D score − 3.00	D score 0.9999	yes phyloP: 0.91	1.Helicase C terminal domain	RCV000120773.1
p.R1093*	*RAD50*	stopgain	C	T	0	0	0.000001	-	-	-	D score 0.9999	yes phyloP: 0.84	1.DNA repair prtoien Rad50 2.P-loop containing nucleoside triphosphate hydrolase	RCV000006230.1
p.L694*	*FANCM*	stopgain	T	G	0	0	0	-	-	-	D score 0.9999	yes phyloP: 0.99	1.ERCC4, restriction endonuclease type II-like,	no data
Splice site mutation														
								Human Splicing Finder	MaxEntScan	Splice Site Finder-like	Mutation Taster			
p.V738L	*EXO1*	splice site	G	C	0	0	0.00000086	D score 93.10	D score 8.44	D score 91.77	D score 0.9999	yes phyloP: 0.89	-	no data

*Poly-Phen2: in the study, we used scores generated by the HumVar trained model as it is preferred for the diagnosis of Mendelian diseases. The scores range from 0 to 1, and a substitution with larger score has a higher possibility to damage the protein function. The program evaluates an amino acid substitution as D = probably damaging (if the score is in the range 1-0.909), P = possibly damaging (score is in the range 0.908-0.447), B = benign (score ≤ 0.446)

*SIFT:* SIFT The amino acid substitution is predicted to be D = damaging if the score is <0.05, otherwise it is predicted as T = tolerated

*FATHMM*: The amino acid substitution is predicted to be D = damaging if the score is < −1.5; otherwise SNP is predicted to be T = tolerated

*MutationTaster*: The scores range from 0 to 1. The larger score suggests a higher probability to cause a human disease. The program output is A = disease causing automatic, *D* = disease causing, N = polymorphism, P = polymorphism automatic

Due to the lack or low frequency of candidate variants in control groups, the estimation of cancer risk associated with the presence of identified variants was not possible. DNA testing of probands’ parents confirmed parental origin of all selected variants. The presence of identified changes in tumor tissue had been proven for *MSH2* p.V606I and *RAD50* p.R1093* variants. For *MSH2* p.A733T variant this analysis was not possible because of the lack of tumor tissue.

Within the prioritized variants only p.R1093* in *RAD50* gene (rs121912628) was reported as pathogenic in ClinVar database (OMIM: 604,040.0001) and in the COSMIC list of variants that have previously been associated with cancer predisposition (COSM1060699) [37].

### *NBN* c.511A>G and c.657_661del5 variants analysis

In 102 patients with *medulloblastoma* six carriers of *NBN* germline likely pathogenic variants (c.511A>G or c.657_661del5) were identified. Additional four *NBN* carriers reported in our previous study [19] were included in the analysis to increase a number of patients for assessment of clinical relevance of detected *NBN* variants.

### Whole exome sequencing analysis

In three patients with severe treatment complications but no presence of *MSH2*, *RAD50* and *NBN* candidate variants the exome sequence was analyzed. In all cases rare candidate variants in genes essential for DNA repair pathway were detected, including p.L694* in *FANCM* (ID 57697, MIM:609,644), p.R695C in *ERCC2* (ID 2068, MIM:126,340) and p.V738L in *EXO1* (ID 9156, MIM:606,063). None of identified variants was reported in 1000 Genomes Database and in matched control group. Two variants p.R695C in *ERCC2* and p.V738L in *EXO1* were found in ExAC Database with frequency 0.000000012 and 0.00000086, respectively. All detected variant were present in heterozygous state (Table 2).

### Characteristics of patients and tumors with molecular variants in DNA repair genes

Distribution of clinical and biological features in patients with molecular variants in essential for DNA repair pathway genes is presented in Table 3.

**Table 3:** The molecular and clinical characteristics of the carriers of DNA repair genes alterations

Patient ID	Identified variant/gene	Metastases	Histologic subtype	Molecular subgroup	Treatment Protocol	Status^a^	Rare adverse events grade 4 on chemotherapy	Drug in the regimen during the incidence of rare adverse events grade 4	The course of chemotherapy
1.	p.V606I/*MSH2*	YES	classic	4	HR	ADF	NO	-	-
2.	p.A733T/*MSH2*	YES	LCA	Non-WNT/SHH (3 or 4)	For relapse	DoD	Pneumonia	Irinotecan, Dacarbazine	pre-irradiation chemotherapy
3.	p.R1093* /*RAD50*	NO	classic	3	HR	ADF	Central nervous system toxicity	Etoposide, Ifosphamide, Cisplatin	pre-irradiation fourth course of chemotherapy
4.	p.Lys219fs*19/*NBN*	YES	classic	4	HR	ADF	no data	-	-
5. ^b^	p.Lys219fs*19/*NBN*	NO	classic	WNT	HR	ADF	NO	-	-
6. ^b^	p.Lys219fs*19/*NBN*	YES	classic	no data	<3 yrs	DoD	Pneumonia	Vincristin, Etoposide, Cisplatin	first course of chemotherapy
7.	p.Lys219fs*19/*NBN*	YES	LCA	4	HR	DoD	NO but secondary AML M5 4 yrs. after diagnosis	-	-
8. ^b^	p.Lys219fs*19/*NBN*	NO	classic	Non-WNT/SHH (3 or 4)	SR	ADF	NO	-	-
9.	p.Lys219fs*19/*NBN*	NO	classic	4	HR	DoD	NO	-	-
10.	p.I171V/*NBN*	YES	classic	4	HR	ADF	NO	-	-
11.	p.I171V/*NBN*	NO	classic	no data	<3 yrs	DoD	Central nervous system toxicity	Vincristin, Etoposide, Endoksane, Cisplatin	after two courses of chemotherapy
12. ^b^	p.I171V/*NBN*	YES	classic	no data	HR	DoD	Colitis with gastrointestinal bleeding/	Vincristin, Etoposide, Carboplatin	pre-irradiation first course of chemotherapy
13.	p.I171V/*NBN*	NO	classic	4	HR	ADF	NO	-	-
14.	p.L694*/*FANCM*	YES	LCA	4	HR	ADF	Central nervous system toxicity	Vincristine, Cisplatin, Lomustin	post radiation chemotherapy
15.	p.R695C/*ERCC2*	NO	classic	Non-WNT/SHH (3 or 4)	SR	ADF	Colitis with gastrointestinal bleeding/	Vincristine, Etoposide, Carboplatin	pre-irradiation first course of chemotherapy
16	p.V738L/*EXO1*	NO	classic	4	SR	ADF	Enterocolitis	Vincristine, Etoposide, Carboplatin	pre-irradiation first course of chemotherapy

Abbreviations: *ADF alive disease free, *DoD* died of disease, ^b^patients reported previously [19] clinical data updated;

*HR- PPNG* High Risk protocol, *SR- PPNG* Standard Risk protocol (Additional file 2: Figure S1A), <3 yrs. – PPNG protocol for children younger than 3 years old (Additional file 2: Figure S1B); LCA - large cell/anaplastic

There were no differences between groups of patients with presence vs. absence of either of candidate variants in *MSH2*, *RAD50* and *NBN* genes in terms of age (<3 years of age vs. ≥ 3 years, not significant, n.s), gender (n.s), LCA pathology (n.s) and presence of metastases (M2 M3 vs. M0 M1, n.s). Tumours belonged to WNT Group (one tumour), Group 3 (one tumour) and Group 4 (8 tumours). Three tumours were not analyzed by NanoString method due to the lack of RNA but they belonged to non-WNT/SHH type (Group 3 or 4). In the remaining three cases molecular type was not determined due to the lack of tumor material. It has been noticed that none of the patients with SHH tumors had *MSH2*, *RAD50* or *NBN* variants (9 patients analyzed) but these data did not reach statistical significance when compared to other groups (n.s).

Four out of 12 patients with *MSH2*, *RAD50* and *NBN* molecular variants did not complete treatment protocol because of reduction of the dose of drugs or delays above 100 days due to presence of various degrees of adverse events. These included 4 out of 6 patients who died, therefore we refrained from examination of survival rates. Among them one *NBN* c.657_661del5 carrier died due to secondary leukemia 48 months after diagnosis.

To assess the potential impact of molecular defects on the course of treatment we recorded that three out of 9 patients with presence of *NBN* variants and available clinical data, suffered from rare grade 4 adverse events during chemotherapy after the first course of treatment. These included central nervous system toxicity, pneumonia and colitis with gastrointestinal bleeding. For comparison, among 89 patients without molecular variants in none of *NBN, MSH2* and *RAD50* genes only three patients suffered from similar complications (enterocolitis grade 4, gastrointestinal bleeding grade 4 and central nervous system toxicity grade 4) despite an application of the same PPNG protocol (Additional file 2: Figure S1 A and B) and the difference was significant (*p* = 0.01). Moreover, two out of three patients with identified variants in *MSH2* or *RAD50* also displayed similar grade 4 complications, namely central nervous system toxicity and pneumonia. Therefore, 5 out of 12 patients with defects in either of *MSH2*, *RAD50* and *NBN* genes suffered from rare grade 4 adverse events during chemotherapy and these combined results were even more significant when compared to the control group (*p* = 0.0005) than for *NBN* gene alone. Among patients with detected defects in *MSH2*, *RAD50* and *NBN* genes High Risk arm of the PPNG protocol (HR, Additional file 3: Table S2, Additional file 2: Figure S1 A and B) which included Cisplatin and Ifosfamide was not applied more frequently than in the series of patients from control group without detected defects in those genes (*p* = 0.13). When patients were subdivided according to treatment arm, the results pointing toward more frequent toxicity in patients with identified variants were also statistically significant (*p* = 0.01 for HR arm only, and *p* = 0.01 for protocol for children <3 years old only).

Because the above results indicate association between defects in analyzed DNA repair genes and presence of adverse effects during therapy we performed WES analyses in three patients who suffered from rare grade 4 adverse events but had no abnormalities in *MSH2*, *RAD50* and *NBN* genes. In all three patients candidate variants were detected in *FANCM*, *ERCC2* and *EXO1* genes which are presented in Tables 2 and 3. In summary, taking into account the WES results, among patients with detected variants in DNA repair genes majority of them (8 out of 15; 53%) suffered from rare life-threatening grade 4 toxicity during the course of treatment.

## Discussion

Assuming that the fundamental feature of cancer is genomic instability, functional defects of proteins which are responsible for maintenance of genome integrity by correcting DNA replication errors, should be carcinogenic. It is therefore not surprising that a number of cancer susceptibility genes encode key factors of DNA repair pathways. Recent comprehensive analysis of germline mutations in pediatric cancers pointed to DNA repair genes as the most commonly mutated genes, including *TP53* and *BRCA2* [43]. It is also increasingly clear that defects in DNA repair genes may determine patient’s response to radio and chemotherapy [13, 16, 17]. In view of that we evaluated the potential association between DNA repair defects and treatment related toxicity as well as their potential role as a susceptibility factor for *medulloblastoma*.

The sequence analysis of two well-known repair genes *MSH2* and *RAD50* conducted in large cohort of 102 *medulloblastoma* patients revealed three new germline variants *MSH2* p.V606I and p.A733T as well as *RAD50* p.R1093*. Both the localization and the character of detected variants allow for prediction of their probably pathogenic impact on the encoded proteins what was supported by the results of the in silico analysis (Table 2). The p.V606I and p.A733T substitutions are localized in the crucial DNA mismatch repair protein V (MutSV - aa 619-854, pF00488) in highly (p.V606I- phyloP:4.40) and moderate (p.A733T-phyloP:2.55) conserved amino acid region. MutSV domain contains the dimerization interface and nucleotide-binding site with C-terminal helix-U-turn-helix motif that is critical for MutS function [44]. The *RAD50* p.R1093* variant resulting in premature stop codon has severe consequences on the protein translation and predicts suppression of its protein. All identified variants were uncommon in our patients (1/102; 0.98%). This is consistent with the published data indicating that molecular variants in *MSH2* and *RAD50* in CNS tumors occurred very rarely (5/1637 – 0.31% and 4/1743 – 0.23%, respectively [37]. In support of that, in published recently study of germline mutations in pediatric cancers, including *medulloblastoma, MSH2* and *RAD50* variants were not reported [43]. Due to the lack or low frequency of candidate variants in control groups the estimation of cancer risk associated with their presence was not possible (Table 2). However, deleterious character of detected germline variants, the role of the encoded proteins in DNA repair system and their annotation with genetic syndromes, including NBSLD and CMRDS associated with *medulloblastoma*, make them the potential susceptibility variants for this kind of tumor. The *RAD50* p.R1093* variant was reported as one of two known molecular defects (HGMD CMO92910) responsible for NBSLD. In *medulloblastoma* patients pathogenic variants in *MLH1*, *MSH6* and *PMS2* genes were detected previously [21–24, 43]. Alterations in these genes together with *MSH2* defects lead to CMRDS. Both *MSH2* and *RAD50* encode the crucial components of the DNA repair system. *MSH2* belongs to mismatch repair genes (MMR) while *RAD50* together with *MRE11* and *NBN* constitute the MRN complex responsible for connecting DNA damage detection to DNA repair and cell cycle checkpoint function [44, 45]. Biallelic deficiency in MMR genes had been referred as a molecular cause of increased predisposition to gastrointestinal and hematological malignances, as well as early-onset CNS tumors (especially *glioblastoma;* GBM) [22, 26]. Additionally, the germline heterozygous variants in MMR gene were reported in patients with Turcot syndrome associated with *medulloblastoma* incidence. The molecular variants affecting genes of the MRN complex might also play a role in pediatric tumor development. The evidence that *NBN* heterozygous variants predispose to childhood *acute lymphoblastic leukemia* and *medulloblastoma* was already published [19, 46–48]. All these facts reinforce potential role of DNA repair genes, including *MSH2* and *RAD50* in susceptibility to *medulloblastoma* but detailed mechanistic studies are required to confirm this preliminary hypothesis.

Notwithstanding the role of DNA repair genes in pathogenesis of *medulloblastoma*, it is profoundly important from the clinical perspective that the presence of molecular defects in these genes may have an impact on the course of treatment.

The MRN complex genes, including *MRE11*, *NBN* and *RAD50* are required for double-stand DNA break (DBS) repair via one of the DNA repair system, homologous recombination (HR). Defects in HR system lead to hypersensitivity to agents that produce DSB and topoisomerase inhibitors eg. etoposide [14, 15, 49]. MMR genes remove mispaired nucleotides by the cooperation in mismatch repair system whose defects are associated with hypersensitivity to DNA crosslinks and platinum-based chemotherapeutic agents (eg. mitomycin C and carboplatin) [14, 15, 49].

*Medulloblastoma* treatment protocol (Additional file 2: Figure S1 A and B) includes the drugs mentioned above, specifically platinum-based chemotherapeutic agents (carboplatin, cisplatin), topoisomerase inhibitor etoposide and, in addition, mitotic inhibitor vincristine. Therefore it is very likely that our patients with molecular variants in DNA repair genes may be more prone to complications in recovering from chemotherapy induced DNA damage. They include the patients with variants detected by WES analysis in *ERCC2*, *FANCM* or *EXO1* genes, an essential components of DNA repair systems [14]. All three identified variants (p.R695C, p.L694* and p.V738L, respectively) were localized in highly conserved nucleotide position (phyloP: 0.89-0.99) in crucial for the encoded protein domains (Table 2) and the character of detected variants (nonsense and splice site) strengthens their pathogenic role. Previous functional studies had also linked these variants to increased sensitivity to therapeutic agents. Defects of ERCC2 protein were reported as a cause of faults in the nucleotide excision repair mechanism (NER) which is responsible for removal of variety of helix-distorting DNA lesions, as well as a hypersensitivity to platinum derivatives. The *FANCM* gene is one of the elements of the Fanconi Anemia (FANC) pathway responsible for DNA crosslinks repair, possibly through coordination of three main DNA repair systems: nonhomologus endjoing (NHEJ), homologus recombination (HR) and translesion DNA synthesis (TLS). Loss of function of this system results in sensitivity to DNA crosslinking agents and platinum derivatives [14, 15, 49]. Finally, *EXO1* gene encode a nuclease which cooperates with MRN complex in DSB repair via HR pathway as well as interacts with MMR genes in repair of DNA mismatches [50, 51].

Although we acknowledge that functional studies are necessary to explore the mechanism through which DNA repair gene defects influence the treatment related toxicity we have already found significant association between defects in *NBN*, *MSH2*, *RAD50*, *FANCM*, *ERCC2* and *EXO1* genes and clinical data. Indeed, more than half of patients with variants in DNA repair genes suffered from rare adverse grade 4 events after administration of chemotherapy (Table 3). We acknowledge that validation cohorts would be necessary for confirmation of our results. Unfortunately, recently published NSG ‘discovery sets’ of *medulloblastoma* ranged only from 39 to 92 samples and molecular defect in *MSH2*, *RAD50* and *NBN* gene were not identified [7, 9–12, 43]. Also, an information related to the therapy and accompanied side effects was not provided in these studies. However in two pediatric *medulloblastoma* patients with mutations in DNA repair genes (*PALB2* and *BRCA2*) chemotherapy inducted grade 4 side effects were reported [16, 17]. In addition, effect of other drugs being introduced to *medulloblastoma* treatment protocols e.g. temozolomide (TZM) may be dependent on the status of mismatch repair genes. In melanoma one variant g.73170T>C in *MSH2* gene (rs2303428) was associated with response and side effects and could be used as a molecular marker for TMZ treatment response [52].

On the other hand it is difficult to compare toxic effects caused by cancer treatment in adult patients harboring defects in DNA repair genes with toxicity observed in still developing and vulnerable tissues in children. Different spectrum of tumors in children and therefore different treatment protocols, including very high doses of drugs, may influence dissimilar reaction in the latter population.

## Conclusions

Our study was conducted in a single institution on the largest series of uniformly treated patients which provided primary data indicating possible link between defects in DNA repair genes and treatment related toxicity in children. Given the complexity of the data in relation to the rarity of *medulloblastoma*, our results needs to be confirmed in independent cohorts. If proven, the special vigilance during and after treatment of patients with pathogenic variants in DNA repair genes should be required. Also additional screening for the presence of molecular variants in patients with manifestation of severe adverse events is necessary to acquire more information about their impact on the course of treatment. Finally, the potential revision of the mode of treatment should be considered for patients with germline defects in DNA repair genes in the future.

## Additional files

Additional file 1: Table S1. The list of *MSH2* and *RAD50* gene germline variants detected in cohort of 102 MB patients. (DOC 92 kb)

Additional file 2: Figure S1A. Polish Pediatric Neurooncology Group (PPNG) treatment protocol for *medulloblastoma* patients (in children older than 3 years). **Figure S1B.** Polish Pediatric Neurooncology Group (PPNG) treatment protocol for *medulloblastoma* patients (in children younger than 3 years). (ZIP 575 kb)

Additional file 3: Table S2. The molecular and clinical characteristics of 102 patients with *medulloblastoma. (XLSX 14 kb)*

## Abbreviations

<3 yrs.: Polish Pediatric Neurooncology Group protocol for children younger than 3 years old
ADF: Alive disease free
AE: Rare life-threatening adverse events
BASC: BRCA1-associated genome surveillance complex
CMHI: Children’s Memorial Health Institute in Warsaw, Poland
CMRDS: Constitutional Mismatch Repair Deficiency Syndrome
COSMIC database: Catalogue of Somatic Mutations
CTC: The Common Toxicity Criteria
D/N: Desmoplastic/nodular type of *medulloblastoma*
DBS: Double-stand DNA break repair
DoD: Died of disease
FANC: Fanconi Anemia
HGMD: Human Gene Mutation Database
HR: Homologous recombination
HR: Polish Pediatric Neurooncology Group High Risk protocol
IHC: Immunohistochemical analyses
Indels: Coding sequence insertion/deletions
LCA: Large cell/anaplastic type of *medulloblastoma*
MAF: Minor allele frequency
MBEN: *Medulloblastoma* with extensive nodularity
MBL: *Medulloblastoma*, subtype not known
MMR: Mismatch repair genes
NBSLD: Nijmegen Breakage Syndrome-like Disorder
NER: Nucleotide excision repair mechanism
NHEJ: Nonhomologus endjoing
OMIM: Online Mendelian Inheritance in Man
PCR: Polymerase Chain Reaction
PPNG: Polish Pediatric Neurooncology Group
SHH: Sonic Hedgehog type of *medulloblastoma*
SR: Polish Pediatric Neurooncology Group Standard Risk protocol
TLS: Translesion DNA synthesis
WES: Whole Exome Sequencing
WNT: Wingless type of *medulloblastoma*
