# Supplementary material for: The germline variants in DNA repair genes in pediatric medulloblastoma: a challenge for current therapeutic strategies
Source: BMC Cancer. 2017 Apr 4;17:239. doi: 10.1186/s12885-017-3211-y (PMC5379555; doi:10.1186/s12885-017-3211-y)
Supplement: Supplementary file 1 — The list of MSH2 and RAD50 gene germline variants detected in cohort of 102 MB patients. (DOC 92 kb) [file 12885_2017_3211_MOESM1_ESM.doc]

**Supplementary Table 1.** The list of *MSH2* and *RAD50* gene germline variants detected in cohort of 102 MB patients.

| **Chromosomal position (GRCh37-hg19)** | **Reference SNP ID (rs)** | **Gene** | **Wild type nucleotide** | **Altered nucleotide** | **Effect on gene function** | **Numer of variants** |
| --- | --- | --- | --- | --- | --- | --- |
| 47630550 | [rs2303426](http://www.ncbi.nlm.nih.gov/projects/SNP/snp_ref.cgi?rs=2303426) | *MSH2* | C | G | intronic | 55 |
| 47637517 | . | *MSH2* | C | T | intronic | 1 |
| 47641559 | . | *MSH2* | TAA | T | splicing | 12 |
| 47641627 | . | *MSH2* | C | C | intronic | 2 |
| 47643457 | [rs4987188](http://www.ncbi.nlm.nih.gov/projects/SNP/snp_ref.cgi?rs=4987188) | *MSH2* | G | G | missense | 4 |
| 47656801 | [rs2347794](http://www.ncbi.nlm.nih.gov/projects/SNP/snp_ref.cgi?rs=2347794) | *MSH2* | G | G | intronic | 52 |
| 47656813 | [rs190832181](http://www.ncbi.nlm.nih.gov/projects/SNP/snp_ref.cgi?rs=190832181) | *MSH2* | G | G | intronic | 1 |
| 47656871 | [rs17224360](http://www.ncbi.nlm.nih.gov/projects/SNP/snp_ref.cgi?rs=17224360) | *MSH2* | T | T | intronic | 4 |
| 47672900 | [rs17224444](http://www.ncbi.nlm.nih.gov/projects/SNP/snp_ref.cgi?rs=17224444) | *MSH2* | C | T | intronic | 2 |
| 47693768 | [rs186239388](http://www.ncbi.nlm.nih.gov/projects/SNP/snp_ref.cgi?rs=186239388) | *MSH2* | T | T | intronic | 1 |
| 47693788 | [rs12998837](http://www.ncbi.nlm.nih.gov/projects/SNP/snp_ref.cgi?rs=12998837) | *MSH2* | A | T | intronic | 22 |
| 47693849 | [rs63750330](http://www.ncbi.nlm.nih.gov/projects/SNP/snp_ref.cgi?rs=63750330) | *MSH2* | T | T | synonymous | 1 |
| 47693959 | [rs3732183](http://www.ncbi.nlm.nih.gov/projects/SNP/snp_ref.cgi?rs=3732183) | *MSH2* | G | G | intronic | 45 |
| 47698108 | [rs61756466](http://www.ncbi.nlm.nih.gov/projects/SNP/snp_ref.cgi?rs=61756466) | *MSH2* | T | T | synonymous,splicing | 2 |
| 47702220 | . | *MSH2* | G | G | missense | 1 |
| 47702451 | . | *MSH2* | GT | G | intronic | 64 |
| 47702458 | [rs201999276](http://www.ncbi.nlm.nih.gov/projects/SNP/snp_ref.cgi?rs=201999276) | *MSH2* | T | T | intronic | 1 |
| 47702459 | . | *MSH2* | T | T | intronic | 1 |
| 47703290 | [rs10495944](http://www.ncbi.nlm.nih.gov/projects/SNP/snp_ref.cgi?rs=10495944) | *MSH2* | G | A | intronic | 1 |
| 47703379 | [rs2059521](http://www.ncbi.nlm.nih.gov/projects/SNP/snp_ref.cgi?rs=2059521) | *MSH2* | C | T | intronic | 86 |
| 47703500 | [rs2303428](http://www.ncbi.nlm.nih.gov/projects/SNP/snp_ref.cgi?rs=2303428) | *MSH2* | T | T | intronic | 18 |
| 47703697 | . | *MSH2* | G | G | missense | 1 |
| 47705448 | . | *MSH2* | T | T | synonymous | 2 |
| 47707806 | . | *MSH2* | C | T | intronic | 1 |
| 47707955 | . | *MSH2* | C | T | missense | 1 |
| 47709796 | . | *MSH2* | TA | T | intronic | 1 |
| 47709806 | [rs17218662](http://www.ncbi.nlm.nih.gov/projects/SNP/snp_ref.cgi?rs=17218662) | *MSH2* | A | T | intronic | 1 |
| 47709807 | [rs6737098](http://www.ncbi.nlm.nih.gov/projects/SNP/snp_ref.cgi?rs=6737098) | *MSH2* | T | T | intronic | 5 |
| 47709835 | . | *MSH2* | G | G | intronic | 1 |
| 131892979 | [rs4526098](http://www.ncbi.nlm.nih.gov/projects/SNP/snp_ref.cgi?rs=4526098) | *RAD50* | G | A | UTR5 | 86 |
| 131911425 | [rs74769721](http://www.ncbi.nlm.nih.gov/projects/SNP/snp_ref.cgi?rs=74769721) | *RAD50* | G | G | intronic | 1 |
| 131915022 | [rs28903086](http://www.ncbi.nlm.nih.gov/projects/SNP/snp_ref.cgi?rs=28903086) | *RAD50* | G | G | missense | 1 |
| 131915213 | [rs17166050](http://www.ncbi.nlm.nih.gov/projects/SNP/snp_ref.cgi?rs=17166050) | *RAD50* | G | G | intronic | 41 |
| 131915279 | [rs75639632](http://www.ncbi.nlm.nih.gov/projects/SNP/snp_ref.cgi?rs=75639632) | *RAD50* | A | G | intronic | 1 |
| 131915317 | [rs2522403](http://www.ncbi.nlm.nih.gov/projects/SNP/snp_ref.cgi?rs=2522403) | *RAD50* | T | T | intronic | 26 |
| 131923330 | . | *RAD50* | G | G | missense | 1 |
| 131923710 | [rs28903091](http://www.ncbi.nlm.nih.gov/projects/SNP/snp_ref.cgi?rs=28903091) | *RAD50* | G | G | missense | 2 |
| 131924341 | [rs104895045](http://www.ncbi.nlm.nih.gov/projects/SNP/snp_ref.cgi?rs=104895045) | *RAD50* | C | T | intronic | 1 |
| 131924421 | [rs146370443](http://www.ncbi.nlm.nih.gov/projects/SNP/snp_ref.cgi?rs=146370443) | *RAD50* | G | G | missense | 1 |
| 131925174 | [rs56798121](http://www.ncbi.nlm.nih.gov/projects/SNP/snp_ref.cgi?rs=56798121) | *RAD50* | A | C | intronic | 3 |
| 131925187 | [rs2706362](http://www.ncbi.nlm.nih.gov/projects/SNP/snp_ref.cgi?rs=2706362) | *RAD50* | T | T | intronic | 3 |
| 131925240 | . | *RAD50* | A | T | intronic | 2 |
| 131939497 | [rs2706377](http://www.ncbi.nlm.nih.gov/projects/SNP/snp_ref.cgi?rs=2706377) | *RAD50* | A | G | intronic | 1 |
| 131939904 | [rs12187537](http://www.ncbi.nlm.nih.gov/projects/SNP/snp_ref.cgi?rs=12187537) | *RAD50* | T | T | intronic | 10 |
| 131944964 | [rs57395760](http://www.ncbi.nlm.nih.gov/projects/SNP/snp_ref.cgi?rs=57395760) | *RAD50* | C | CT | intronic | 1 |
| 131953722 | . | *RAD50* | C | G | intronic | 1 |
| 131953874 | [rs121912628](http://www.ncbi.nlm.nih.gov/projects/SNP/snp_ref.cgi?rs=121912628) | *RAD50* | C | T | stopgain | 1 |
| 131972916 | [rs137906075](http://www.ncbi.nlm.nih.gov/projects/SNP/snp_ref.cgi?rs=137906075) | *RAD50* | A | G | intronic | 19 |
| 131973002 | [rs56308622](http://www.ncbi.nlm.nih.gov/projects/SNP/snp_ref.cgi?rs=56308622) | *RAD50* | A | G | intronic | 1 |
| 131973177 | [rs2040704](http://www.ncbi.nlm.nih.gov/projects/SNP/snp_ref.cgi?rs=2040704) | *RAD50* | A | G | intronic | 1 |
| 131973290 | . | *RAD50* | T | C | intronic | 1 |
| 131973382 | [rs6872131](http://www.ncbi.nlm.nih.gov/projects/SNP/snp_ref.cgi?rs=6872131) | *RAD50* | A | G | intronic | 1 |
| 131977992 | . | *RAD50* | A | ACAT | nonframeshift-insertion | 1 |
