# Supplementary figures and images for: The germline variants in DNA repair genes in pediatric medulloblastoma: a challenge for current therapeutic strategies
Source: BMC Cancer. 2017 Apr 4;17:239. doi: 10.1186/s12885-017-3211-y (PMC5379555; doi:10.1186/s12885-017-3211-y)

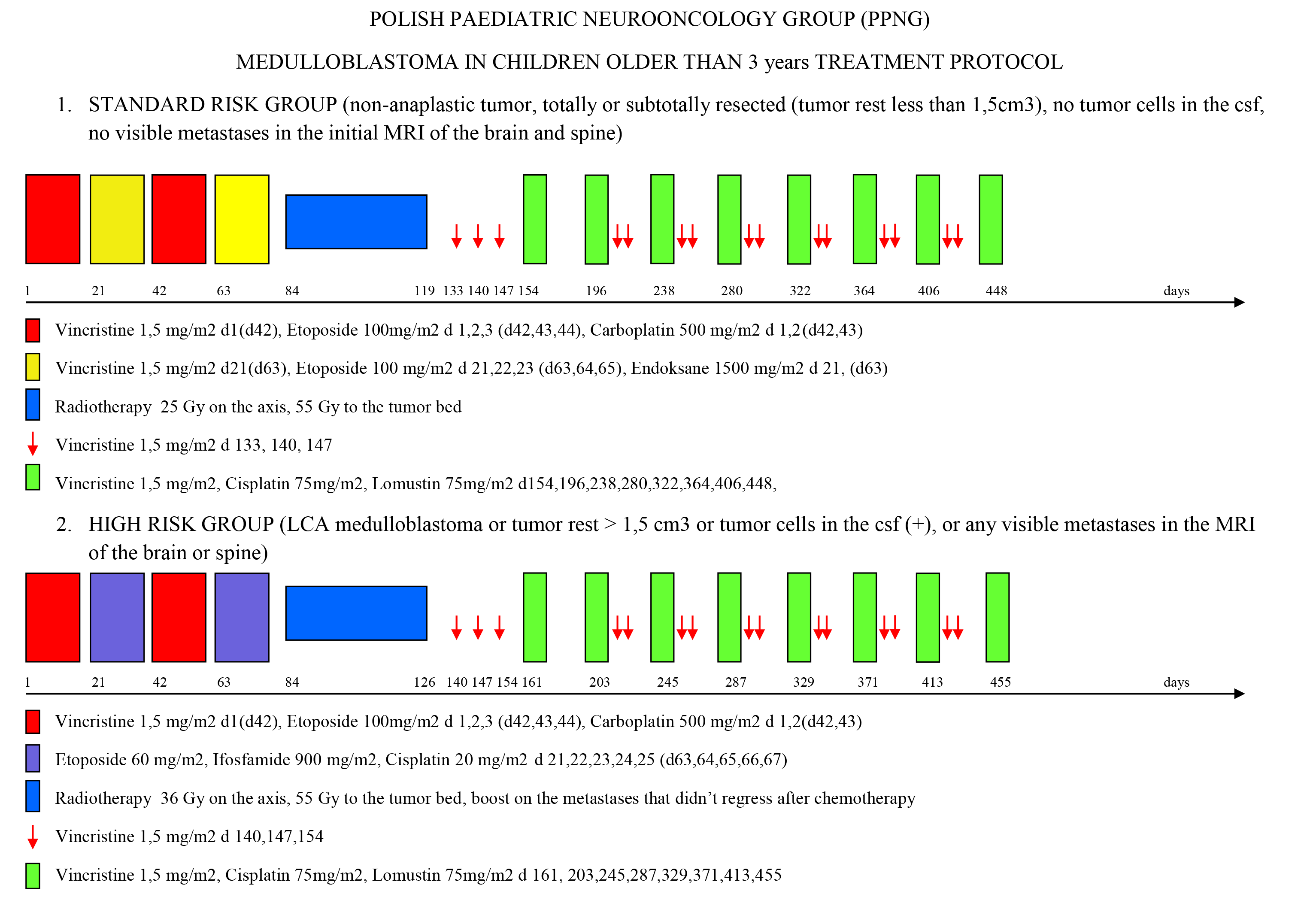

Supplement: Supplementary file 2 — Polish Pediatric Neurooncology Group (PPNG) treatment protocol for medulloblastoma patients (in children older than 3 years). Figure S1B. Polish Pediatric Neurooncology Group (PPNG) treatment protocol for medulloblastoma patients (in children younger than 3 years). (ZIP 575 kb) [file 12885_2017_3211_MOESM2_ESM.zip › Supplementary Figure 1AR3.tif]

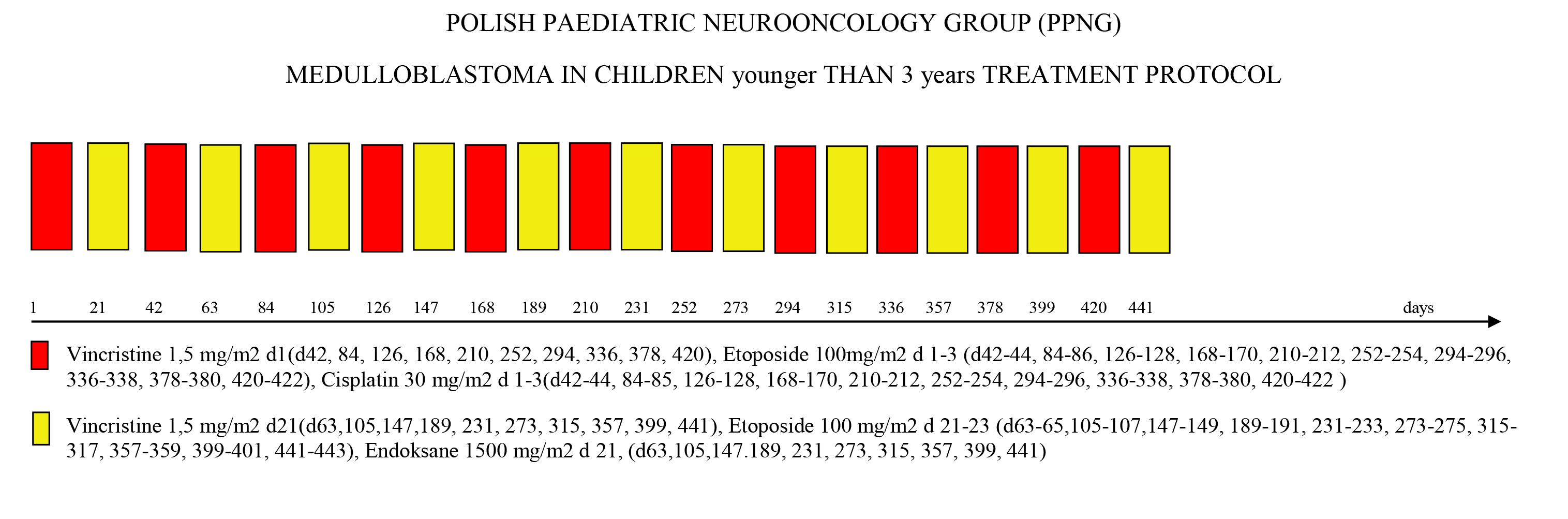

Supplement: Supplementary file 2 — Polish Pediatric Neurooncology Group (PPNG) treatment protocol for medulloblastoma patients (in children older than 3 years). Figure S1B. Polish Pediatric Neurooncology Group (PPNG) treatment protocol for medulloblastoma patients (in children younger than 3 years). (ZIP 575 kb) [file 12885_2017_3211_MOESM2_ESM.zip › Supplementary Figure 1BR3.tif]
